# Asymmetric Type F Botulism with Cranial Nerve Demyelination

**DOI:** 10.3201/eid1801.110471

**Published:** 2012-01

**Authors:** Alina Filozov, Jessica A. Kattan, Lavanya Jitendranath, C. Gregory Smith, Carolina Lúquez, Quyen N. Phan, Ryan P. Fagan

**Affiliations:** Middlesex Hospital, Middletown, Connecticut, USA (A. Filozov, L. Jitendranath);; Centers for Disease Control and Prevention, Atlanta, Georgia, USA (J.A. Kattan, C. Lúquez, R.P. Fagan);; Connecticut Department of Public Health, Hartford, Connecticut, USA (J.A. Kattan, Q.N. Phan);; North Carolina Department of Health and Human Services, Raleigh, North Carolina, USA (C.G. Smith)

**Keywords:** botulism, bacteria, demyelinating disease, neurology, cranial nerves, autopsy, pathology

## Abstract

We report a case of type F botulism in a patient with bilateral but asymmetric neurologic deficits. Cranial nerve demyelination was found during autopsy. Bilateral, asymmetric clinical signs, although rare, do not rule out botulism. Demyelination of cranial nerves might be underrecognized during autopsy of botulism patients.

Botulism is an illness caused by neurotoxin-producing *Clostridium* species (*1**,**2*). Botulinum neurotoxins are classified into 7 types, A–G. Toxin types A, B, and E cause most human botulism, and type F represents only 1% of reported cases (*1**,**2*). The source of intoxication with type F botulism among adults is often uncertain but might result from either intestinal colonization with clostridial spores and subsequent intraintestinal toxin production or from ingestion of preformed toxin in contaminated food (*1*). Antimicrobial drug use and functional or anatomic bowel abnormalities can facilitate colonization through alteration of normal gut flora (*1**,**3*).

The clinical signs of botulism typically consist of bilateral, symmetric cranial nerve palsies and descending, symmetric, flaccid paralysis (*2**,**3*). Illness from type F botulism is distinguished by a fulminant onset and short duration (*1**,**4*). We report atypical type F botulism associated with demyelination of cranial nerves.

## The Patient

While traveling in Connecticut, a man from North Carolina in his mid-60s was admitted to a community hospital for new onset of diplopia, vertigo, truncal ataxia, and vomiting. Approximately 10 days before admission, the patient had been prescribed doxycycline for sinusitis. The physical examination at admission was notable for dilated, asymmetric (5 mm on the right and 4 mm on the left), sluggishly reactive pupils; cranial nerve IV palsy; bilateral proptosis (right more than left); bilateral peripheral facial weakness; and proximal left upper extremity weakness. Routine blood test results and imaging studies of head, brain, and orbits were unremarkable.

During hospital day 1, the patient experienced hypophonia, complete ophthalmoplegia, bilateral ptosis (right more than left), pupils unresponsive to light, dysphagia, and bilateral limb-girdle muscular weakness. His cognition remained intact, and no sensory deficits were documented. Cerebrospinal fluid contained 4 leukocytes/mm^3^, 70 mg glucose/dL, and 43 mg protein/dL; bacterial cultures were negative. Serum was negative for IgG and IgM against *Borrelia burgdorferi*, and rapid plasma reagin test results were negative. Test results for antibody levels against *Campylobacter* spp. and gangliosides (anti-GQ1b IgG) were negative.

On hospital day 2, the patient had difficulty breathing and bulbar signs progressed, followed by descending extremity weakness (left more than right) and areflexia. A diagnosis of Miller Fisher syndrome (MFS), a variant of Guillain-Barré syndrome, was considered, and treatment with intravenous immunoglobulin was begun.

The Connecticut Department of Public Health and the Centers for Disease Control and Prevention (CDC) were contacted for a botulism consultation. Botulinum antitoxin was not administered at that time because asymmetric neurologic deficits and lack of exposure to injection-drug use or home-preserved foods made botulism unlikely. Respiratory paralysis progressed, and on hospital day 3 the patient required mechanical ventilation. A diagnosis of botulism was reconsidered; however, antitoxin was not administered because an alternative diagnosis (MFS) was still likely. Serum was collected on hospital day 5 and sent to CDC for botulism testing; a stool sample was collected on hospital day 14 after resolution of ileus. Neurologic improvement was first noted on hospital day 7, consisting of improved upper-extremity strength. On hospital day 14, weaning from mechanical ventilation was complete.

Botulinum toxin type F was confirmed in the serum sample on hospital day 16. Treating physicians and CDC agreed that administration of antitoxin might still be beneficial because of potential clostridial intestinal colonization. On hospital day 17, investigational heptavalent (A–G) botulinum antitoxin was administered (*5*). Stool sample was negative for botulinum toxin and botulinum toxin–producing *Clostridium* spp. On hospital day 28, the patient experienced self-limited serum sickness. He was discharged to a rehabilitation center later the same day.

At the time of admission to the rehabilitation center, the patient was able to stand with assistance. At 6 weeks after symptom onset, he was able to keep his eyes open and walk with assistance, but dysphagia persisted. Subsequently, he continued to improve. Approximately 9 weeks after the illness had begun, the patient was found unresponsive; cardiopulmonary resuscitation was unsuccessful. Autopsy was limited to the brain and demonstrated inflammatory demyelination of cranial nerve tissue (Figure).

**Figure:**
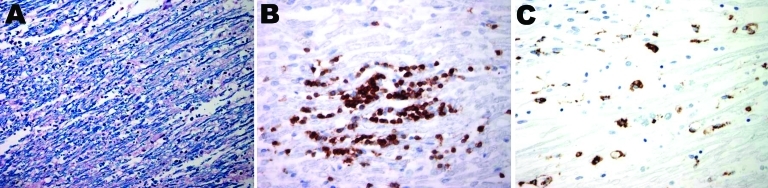
Postmortem cranial nerve tissue from a patient with botulism. A) Fragmentation of myelin sheaths and inflammatory infiltration of B and CD3^+^ T-cells within the nerve tissue (original magnification ×200). B) B-cell infiltration of nerve tissue; C) CD68-positive myelinoklastic macrophages (original magnification ×400).

An epidemiologic investigation was conducted by state and local health departments in Connecticut and North Carolina. No contacts experiencing similar paralytic illness were identified. Two food items consumed by the patient were submitted for CDC analysis; both were negative for botulinum toxin and botulinum toxin–producing *Clostridium* spp*.*

## Conclusions

This case illustrates the challenge of diagnosing a rare form of botulism in a patient with atypical clinical features. At initial examination, the patient had bilateral but asymmetric cranial nerve deficits and extremity weakness. Asymmetric clinical signs are unusual for botulism but have been documented previously with non–type F botulism (*6*). Additionally, truncal ataxia, an uncommon finding, was present. The time from symptom onset to intubation for this patient was 3 days, which is longer than previously recorded for type F patients (most are intubated within <24 hours) (*1*). Some clinical characteristics were typical for type F botulism, such as time to initial motor improvement and duration of ventilatory support (*1*). Also similar to reports of other type F cases, t*he mechanism of botulism intoxication in this patient was unclear (*1*). Intestinal colonization was suspected on the basis of recent antimicrobial drug use and absence of known risk factors for foodborne or wound botulism but was not thoroughly investigated because of the limited availability of stool samples.*

A diagnosis of MFS was considered early in the clinical presentation but was eventually ruled out in favor of botulism; botulism sometimes is misdiagnosed as MFS (*7*). The triad of ophthalmoplegia, areflexia, and ataxia in this patient supported a diagnosis of MFS (*8*), although the former 2 findings also can be observed with botulism (*3*). Progression to descending paralysis was typical of botulism (*2**,**3*). The cerebrospinal fluid protein level and *Campylobacter* spp. and anti-GQ1b IgG ganglioside antibody test results did not support a diagnosis of MFS. Anti-GQ1b ganglioside antibodies are present among >90% of MFS patients (*8*). Given the descending pattern of paralysis, positive mouse bioassay for type F botulinum neurotoxin, and lack of supporting laboratory evidence for an MFS diagnosis, we believe that the patient’s neurologic illness was caused by botulism alone.

The patient’s cause of death is unclear. Death occurred after 2 months of sustained neurologic recovery; botulism relapse was not clinically apparent. Brain autopsy did not elucidate a cause of death; however, the cranial nerve demyelination is noteworthy. According to rare reports, neuropathologic features of botulism include normal histopathologic appearance of peripheral nerves and nonspecific, microscopic hemorrhage and vascular engorgement in the central nervous system (*9**,**10*). However, cranial nerve demyelination was reportedly found in 1 type A botulism patient who received type E antitoxin (*11*). The mechanisms that account for termination of botulinum toxin action and elimination of toxin from cranial nerves remain unidentified, and the possibility of toxin-induced demyelination cannot be excluded in the patient reported here. Alternatively, the abundant inflammatory cells in areas of demyelination might reflect the allergic response to investigational heptavalent botulinum antitoxin manifested by serum sickness reaction. Although we are unable to conclude which hypothesis is more likely, the fact that the patient’s baseline neurologic function was within normal limits weighs against causes preceding his episode of botulism.

We conclude that a bilateral but asymmetric presentation of neurologic signs, although rare, does not rule out the possibility of botulism. In additional, demyelination of cranial nerves might be an underrecognized finding during autopsy of botulism patients, possibly resulting from either the effects of botulism or its treatment.
